# Abdominal-Pelvic Actinomycosis Mimicking Malignant Neoplasm

**DOI:** 10.1155/2011/747059

**Published:** 2011-08-29

**Authors:** Teresa Pusiol, Doriana Morichetti, Corrado Pedrazzani, Francesco Ricci

**Affiliations:** ^1^Section of Cytopathology, Institute of Anatomic Pathology, Rovereto Hospital, 38068 Rovereto, Italy; ^2^Institute of Anatomic Pathology, Rovereto Hospital, 38068 Rovereto, Italy; ^3^Division of Surgery, Rovereto Hospital, 38068 Rovereto, Italy

## Abstract

Abdominal-pelvic actinomycosis is often mistaken for other conditions, presenting a preoperative diagnostic challenge. In a 46-year-old female, computed tomography showed an abdominal-pelvic retroperitoneal mass extending from the lower pole of the right kidney to the lower pelvis. The patient had a 3-year history of intrauterine device. The mass appeared to involve the ascending colon, cecum, distal ileum, right Fallopian tube and ovary, and ureter anteriorly and the psoas muscle posteriorly. The resection of retroperitoneal mass, distal ileum appendicectomy, right hemicolectomy, and right salpingo-oophorectomy was performed. The postoperative period was uneventful. Penicillin therapy was given for six months without any complication. The retroperitoneal mass measured 4.5 × 3.5 × 3 cm, surrounded adjacent organs and histologically showed inflammatory granulomatous tissue, agglomeration of filaments, and sulfur granules of *Actinomyces*, with positive reaction with periodic acid Schiff. Right tubo-ovarian abscess was present. Abdominalpelvic actinomycosis should always be considered in patients with a pelvic mass especially in ones using intrauterine device.

## 1. Introduction

In developed countries, actinomycosis is a relatively rare disease that is mainly caused by *Actinomyces israelii. Actinomyces israelii* is an anaerobic, gram-positive organism that is normally present in oral cavity, throughout the gastrointestinal tract, female genital tract, and the bronchus. Actinomycosis occurs most frequently in the cervical facial (50%–65%), abdominal (20%), and thoracic (15%) regions. The overall incidence of registered cases of actinomycosis is decreasing. Abdominalpelvic actinomycosis, however, are increasing in frequency and is associated with abdominal surgery (such as appendectomy), bowel perforation, or trauma [1]. In addition, the presence of a long-standing intrauterine device (IUD) is a reported risk factor in young women [2]. The abdomen is the most frequent site for actinomycosis and when an abdominal tumor presents as the clinical symptom, the local lesion needs to be differentiated from abdominal tumors of other etiologies, malignancy in particular.

In the majority of cases, the indolent clinical course together with the malignant like tumour appearance at imaging investigations make a delay in diagnosis the rule rather than the exception. Preoperative diagnosis is usually difficult with the majority of cases being diagnosed after the histological and bacteriological examination of the resected specimen. The present paper discusses the case of an abdominalpelvic actinomycosis mimicking a malignant retroperitoneal tumour in a young insulin-dependent diabetic Italian woman with 3-year history of IUD.

## 2. Case Presentation

**Figure 1:**
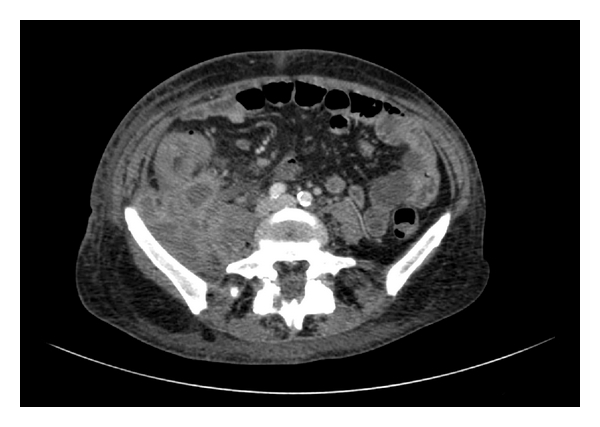
Computed tomography (CT) scan of the abdomen showed the mass occupying the retroperitoneal space and infiltrating the ascending colon.

**Figure 2:**
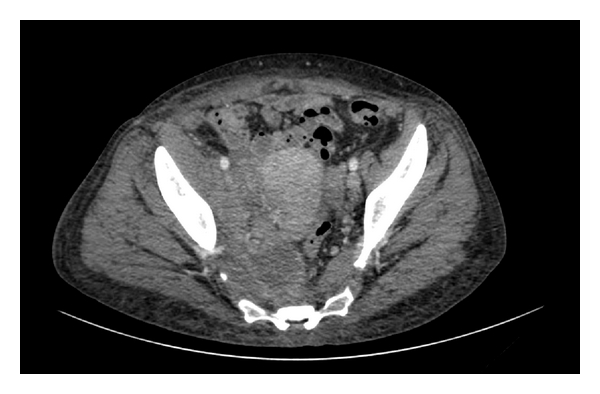
Computed tomography (CT) scan showed the mass occupying the extraperitoneal space infiltrating the psoas muscle posteriorly and the pelvis anteriorly.

A 46-year-old female was referred to our unit following a computed tomography (CT) scan which demonstrated an abdominalpelvic retroperitoneal mass. The patient had came to emergency department complaining of a three-day history of a lump on the right lower limb preceded by fever and continuous right lower abdominal pain irradiated to the back for the previous 3 weeks. Past medical history was unremarkable except for insulin-dependent diabetes mellitus since 11 years of age. The patient had a 3-year history of IUD which had recently been removed. Physical examination demonstrated mild oedema of the right leg, with no abdominal abnormal findings. Doppler ultrasonography of the lower limbs was carried out and ruled deep venous thrombosis and superficial thrombophlebitis. The patient was discharged and she was investigated as an outpatient. Biochemical and haematological investigations demonstrated a raised CRP and ESR, normal white blood count, mild macrocytic anemia (Hb 7.9 g/dL, MCV 100 fL), and thrombocytosis (PLT 626.000/uL). The CT scan showed a retroperitoneal mass with abscess areas and necrosis extending from the lower pole of the right kidney to the lower pelvis. The mass appeared to involve the ascending colon, cecum, distal ileum, right Fallopian tube and ovary, and ureter anteriorly and the psoas muscle posteriorly (Figures 1 and 2). Right ureteric dilatation was evident. A colonoscopy was carried out to investigate the possibility of inflammatory bowel disease or a colonic perforated cancer. The endoscopic examination was normal except for the presence of nonspecific mucosal inflammation of the distal ileum. A US-guided fine needle aspiration biopsy of the mass was hence performed. The cytological specimen showed inflammatory cells, with no evidence of malignant cells. Tuberculous and nontuberculous mycobacterium DNA was also negative. 

 The patient was hence referred to surgery division in the suspect of malignant retroperitoneal mass.

A right ureteric stent was placed and an explorative laparotomy was preformed. The intraoperative findings were compatible with a neoplastic mass originating from the retroperitoneum. Debulking of retroperitoneal, appendicectomy right hemicolectomy extended to the distal ileum, and right salpingo-oophorectomy were performed. The postoperative period was uneventful and the patient was discharged in postoperative day 9. 

Penicillin therapy was given for six months without any complication. She is well and has gained weight after one year.

**Figure 3:**
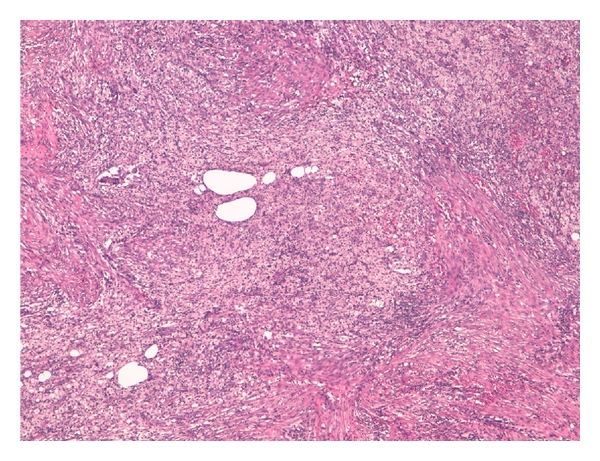
The retroperitoneal mass consisted of chronic suppurative granolomatous inflammation, H&E 20x.

**Figure 4:**
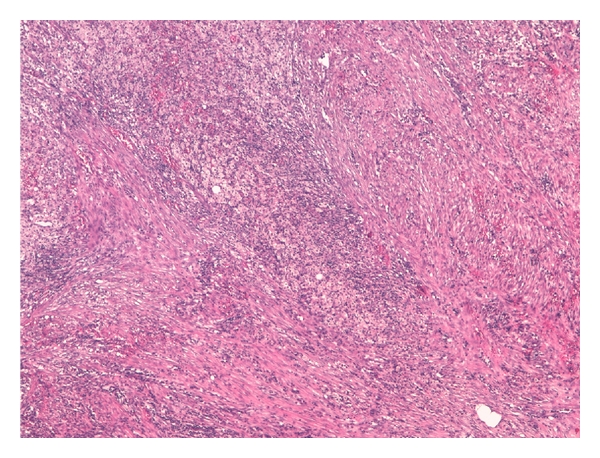
The inflammation was composed by fibroblasts, xanthomatous cells, and neutrophilic granulocytes, H&E 20x.

**Figure 5:**
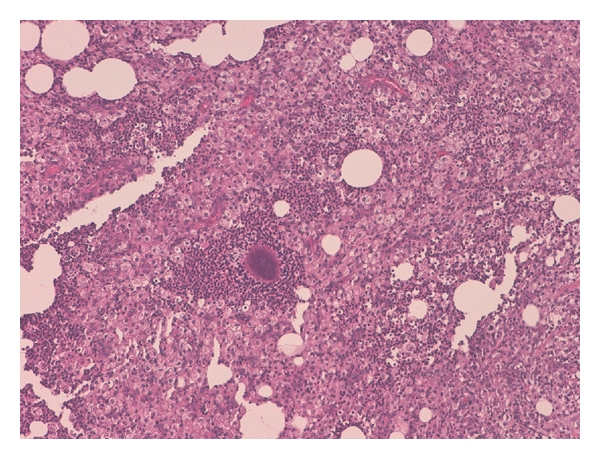
Colonies of *Actinomyces* species were detected with surrounding inflammatory infiltration, H&E 40x.

**Figure 6:**
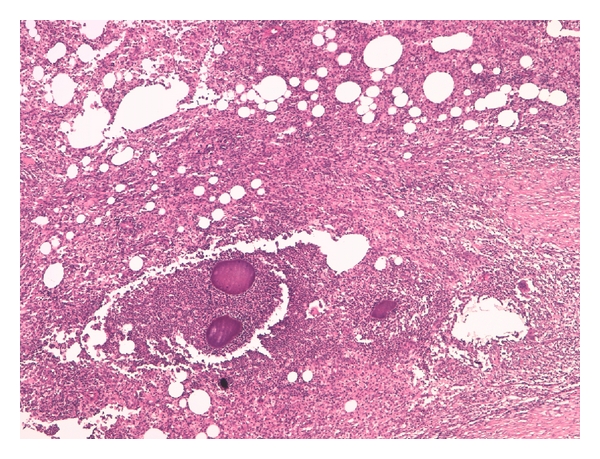
Colonies of *Actinomyces* species were founded in abscess areas, H&E 40x.

The retroperitoneal mass measured 4.5 × 3.5 × 3 cm, surrounded adjacent organs and histologically showed inflammatory granulomatous tissue composed by granulocytes, fibroblasts, xanthomatous cells, and agglomeration of filaments and sulfur granules of *Actinomyces*, with positive reaction with periodic acid-Schiff and Grocott's dye. Abscess formation, necrosis were found (Figures 3, 4, 5 and 6). Similar inflammatory granulomatous process was present in the serosa of terminal ileum, appendix, cecum, ascending colon with extension to corresponding mesentery. Regional 22 lymph nodes were free of disease. Right tubo-ovarian abscess was present. The mucosa of all organs examined did not show actinomycosis but only congestion and slight aspecific inflammation.

## 3. Discussion

*Actinomyces israelii* as other bacteria of the *Actinomyces* species are saprophytes in the oral cavity, gastrointestinal, and female genital tract. The destruction of the muscular barrier by trauma, that is, endoscopic manipulation, operations, immunosuppression, and chronic inflammatory disease, is recognized as predisposing factors for penetration of *Actinomyces* bacteria [3]. Several forms of immunosuppression, such as leukemia, lymphoma, renal insufficiency, renal transplant, and diabetes, have been demonstrated to facilitate this process [4]. It is accepted that the risk of pelvic actinomycosis resulting from IUD use is very low. Only about 92 reported cases exist in the published English language literature, despite 30 million patient-year of IUD use [5]. About 80% of cases of pelvic actinomycosis have been reported in women using an IUD. *Actinomyces israelii* infects 1.65% to 11.6% of IUD users, and infection is more common in women who have had an IUD use in situ longer than four years [6].

Our patient had a 3-year history of IUD which had recently been removed. The IUD may be considered the initial trigger of abdominalpelvic actinomycosis. Ileocecal region and appendix itself are the most frequently involved regions. Recognized causes of infection are appendicitis, diverticulitis, inflammatory bowel disease, and previous open and laparoscopic surgery. Endoscopic procedures have been also described as rare potential causes. No previous surgery or history of inflammatory diseases of the abdomen were reported by our patient.

Clinical symptoms are usually not specific and include a wide range of clinical presentation. Acute abdomen can be observed when complications such as perforation or fistulization occur; more frequently, as in our case, abdominal pain is present.

Preoperative diagnosis of pelvic abdominal actinomycosis can be difficult because of the insidious nature of the infection. Biochemical and haematological investigations are almost not specific. Usually, diagnosis with fine-needle aspiration cytology is in impossible pre-operatively. In fact the filaments and sulfur granules of *Actinomyces* are surrounded by extensive inflammatory tissue that is the sample site of fine-needle aspiration cytology. In our case these procedures were conclusive of inflammatory lesion.

Preoperative radiologic diagnosis is rarely performed. Ha et al. [7] analyzed the CT findings of ten patients with abdominal actinomycosis. The aggressive nature of invasion and infiltration of contiguous tissues and organs, such as the large intestine, greater omentum, or abdominal wall, was remarkable and comparable to that seen in acute necrotizing pancreatitis. Lee et al. [8] have examined CT scans in 18 patients with pathologically proved abdominalpelvic actinomycosis involving the gastrointestinal tract. Eight patients had a history of using IUDs. The sigmoid colon was most commonly involved (50%). All patients showed concentric (*n* = 15) or eccentric (*n* = 3) bowel wall thickening, with a mean thickness of 1.2 cm and a mean length of 8.3 cm. The thickened bowel enhanced homogeneously in nine patients and heterogeneously in the other nine. Inflammatory infiltration was mostly diffuse and severe. In 17 patients, a peritoneal or pelvic mass (mean maximum diameter, 3.2 cm) was seen adjacent to the involved bowel and appeared to be heterogeneously enhanced in most cases; infiltration into the abdominal wall was seen in four patients.

*Actinomycosis* should be included in the differential diagnosis when CT scans show bowel wall thickening and regional pelvic or peritoneal mass with extensive infiltration, especially in patients with abdominal pain, fever, leukocytosis, or long-term use of intrauterine contraceptive devices.

Neoplasms and other inflammatory diseases, especially tuberculosis or Crohn's disease, may be confused with actinomycosis. In actinomycosis, solid masses with focal low-attenuation areas were more frequently found than cystic masses with thickened walls. In conclusion, imaging investigations (US, CT, and MRI) confirm the presence of a mass with collections but they are not able to distinguish between actinomycosis and malignancy, Crohn's disease, diverticulitis, appendicitis, pelvic peritonitis, or tubercolosis [9]. 

The infiltrative mass with unusual aggressiveness is the one of important radiological findings. 

In our case the CT scan showed an infiltrative mass with unusual aggressiveness. The lymph node enlargement, ascites and involvement of the whole peritoneal cavity were absent. These findings could be supported by the diagnosis of Actinomycosis in our case.

Similarly to our case, in the great majority of cases, diagnosis is reached by histopathological examination of the specimen obtained by surgical exploration and resection. Histopathologic examination of the infected tissue should include a search for characteristic, but not pathognomonic, appearances of sulphur granules. The granules measure 0.4–4 mm and stain Gram-positive with a mycelium-like structure [10]. The differential diagnosis of sulphur granules, however, includes nocardiosis, streptomycosis, chromomycosis, eumycetoma, and botryomycosis [11]. *Actinomyces* granules regularly show a positive reaction with periodic acid Schiff and Grocott's dye, but the Kossa reaction is negative. Pseudoactinomyces granules formed by *Nocardia* and *Streptomyces* spp. show the opposite reactions [12]. Because of the size of the bacterium, it usually does not spread via the lymphatic system; therefore, regional lymphadenopathy is uncommon or develops late [13]. In our case the intense proliferation of fibroblasts and xanthomatous cells may be considered the cause of sizes of retroperitoneal mass simulating malignancy. The necrosis and abscess areas have progressively increased the inflammatory mass with compression and infiltration of adjacent organs. The histological examination showed regional lymph nodes free of disease.

## 4. Conclusions

The primary diagnosis of abdominalpelvic actinomycosis is difficult. The clinical picture has changed in the last ten years. Women with IUDs are especially at risk. All organs and anatomic structures of the abdomen can be involved. Even with extensive infection, combined operative and antibiotic therapy allows cure in most cases.
